# Case Report: Case series: growth hormone replacement for severe growth hormone deficiency during BRAF inhibitor therapy in pediatric low-grade glioma: long-term follow-up

**DOI:** 10.3389/fendo.2026.1940850

**Published:** 2026-10-08

**Authors:** Marta Panciroli, Antonio Verrico, Marco Crocco, Sara Cencioni, Chiara Morreale, Antonia Ramaglia, Valentina Iurilli, Gianluca Piccolo, Claudia Milanaccio, Mohamad Maghnie, Natascia Di Iorgi

**Affiliations:** 1 Department of Neuroscience, Rehabilitation, Ophthalmology, Genetics, Maternal and Child Health, University of Genoa, Genoa, Italy; 2 Neuro-Oncology Unit, Istituto di Ricovero e Cura a Carattere Scientifico (IRCCS) Istituto Giannina Gaslini, Genoa, Italy; 3 Pediatric Gastroenterology and Endoscopy Unit, Istituto di Ricovero e Cura a Carattere Scientifico (IRCCS) Istituto Giannina Gaslini, Genoa, Italy; 4 Department of Woman and Child, ASST-Settelaghi, University of Insubria, Varese, Italy; 5 Neuroradiology Unit, Istituto di Ricovero e Cura a Carattere Scientifico (IRCCS) Istituto Giannina Gaslini, Genoa, Italy; 6 Pharmacy, Istituto di Ricovero e Cura a Carattere Scientifico (IRCCS) Istituto Giannina Gaslini, Genoa, Italy; 7 Pediatric Clinic, Endocrinology, Istituto di Ricovero e Cura a Carattere Scientifico (IRCCS) Giannina Gaslini, Genoa, Italy

**Keywords:** body composition, BRAF inhibitor, growth hormone deficiency, growth velocity, pediatric low-grade glioma, recombinant human growth hormone, vemurafenib

## Abstract

Pediatric low-grade gliomas (pLGGs) involving the hypothalamic-pituitary region frequently result in endocrine dysfunction, particularly growth hormone deficiency (GHD), due to tumor location and treatment-related effects. Growth hormone replacement therapy (GHRT) is generally considered after a period of disease stability, whereas its use during active oncologic therapy remains controversial, including during BRAF inhibitor (BRAFi) treatment. We describe two pediatric patients with severe GHD who received GHRT during ongoing vemurafenib, a BRAFi, for progressive chiasmatic-hypothalamic pLGG. The first patient, a girl diagnosed in infancy with desmoplastic infantile ganglioglioma, developed severe GHD after biopsy, chemotherapy, partial tumor debulking, and targeted therapy, together with multiple additional endocrine deficiencies. The second patient, a boy with pilocytic astrocytoma, was diagnosed with severe GHD after multimodal treatment, including partial and subtotal surgical resections, chemotherapy, radiotherapy, and targeted therapy. In both cases, low-dose GHRT was initiated after multidisciplinary assessment, documented radiological stability before GHRT initiation, and family counseling regarding the absence of direct safety data during active BRAFi therapy. Treatment was titrated over two months, with regular IGF-1 monitoring and close endocrine and oncologic surveillance. During approximately six years of concomitant GHRT and vemurafenib exposure, growth stabilized and lipid parameters improved, whereas catch-up growth remained limited and bone outcomes were complex, with increased bone mineral content but persistent or worsening lumbar DXA-derived impairment. No tumor progression or secondary malignancies were detected during the reported follow-up. These two cases add to the limited evidence on GHRT administration during BRAFi therapy by documenting prolonged concomitant GHRT and vemurafenib exposure in children with severe confirmed GHD and previously progressive chiasmatic-hypothalamic pLGG. However, the limited sample size and observational nature of this report preclude conclusions about safety or efficacy. Larger prospective studies are needed to define which patients may benefit and under which monitoring conditions GHRT could be considered during BRAFi therapy.

## Introduction

Pediatric low-grade gliomas (pLGGs) are the most common central nervous system tumors in children, accounting for approximately 30% of pediatric central nervous system (CNS) tumors. Although overall survival is excellent, event-free survival remains relatively low, particularly in non-NF1 patients, and many children require multiple lines of treatment (1–4). Molecular characterization has shown that approximately 90% of pLGGs harbor alterations in the RAS/MAPK pathway, providing a rationale for targeted approaches, including BRAF and MEK inhibition (5, 6). Vemurafenib, an oral selective ATP-competitive inhibitor of BRAFV600E kinase, has demonstrated antitumor activity in recurrent or progressive BRAFV600E-positive brain tumors, with a generally manageable toxicity profile. Since 2015, our institution has offered vemurafenib as a possible treatment for relapsed pLGG carrying the BRAF V600E mutation. Clinical experience with vemurafenib and other BRAF/MEK inhibitors introduced subsequently for treating such patients suggests that prolonged treatment may be required in selected patients to maintain tumor control and reduce the risk of rapid regrowth after discontinuation (7, 8).

Approximately 40% of pLGGs involve the optic pathway and/or hypothalamus, frequently resulting in hypothalamic-pituitary dysfunction. Growth hormone deficiency (GHD) is among the most common endocrine sequelae in this setting and may contribute not only to impaired linear growth, but also to adverse body composition, dyslipidemia, reduced bone mineral accrual, and impaired quality of life (9, 10). Current guidelines recommend considering growth hormone replacement therapy (GHRT) in childhood cancer survivors with confirmed GHD only after an appropriate interval off oncologic treatment and in the presence of stable disease, following multidisciplinary evaluation (11). However, these recommendations do not specifically address children with severe GHD who remain on prolonged targeted therapy for tumor control.

The use of GHRT during active oncologic therapy therefore remains a challenging and poorly defined clinical scenario. This is particularly relevant for children with hypothalamic-chiasmatic pLGG receiving long-term BRAF inhibition, in whom untreated severe GHD may worsen growth, metabolic, and skeletal morbidity, while direct safety data on GHRT during targeted therapy are lacking.

To our knowledge, this is the first report describing two children with severe secondary GHD who received GHRT during ongoing vemurafenib treatment for recurrent/progressive chiasmatic-hypothalamic pLGG. The aim of this case series is to describe the clinical decision-making process, auxological, metabolic, skeletal, and oncologic follow-up, and the limitations of interpreting outcomes in this complex setting.

## Case description

Case 1: A female child with a previously unremarkable medical history was diagnosed with a chiasmatic-hypothalamic ganglioglioma at 5 months of age. Presenting symptoms included blurred vision and bilateral nystagmus. Initial MRI showed an optic pathway/hypothalamic mass without evidence of metastatic spread. After biopsy, the diagnosis of desmoplastic infantile ganglioglioma was established.

First-line treatment was administered according to the European SIOP LGG 2004 protocol, with standard induction using vincristine and carboplatin. Because of refractory disease, she received additional treatments, including the BB-SFOP protocol for 1 year, weekly vinblastine for 4 months, and, due to allergic reactions to carboplatin, vincristine/cisplatin alternating with vincristine/cyclophosphamide according to a modified SIOP LGG 2004 regimen for 1 year (2, 12, 13). Neuroradiological evaluation showed reduction of the anterior tumor component, whereas the posterior lesion progressed. At 4.8 years of age, partial debulking of the posterior lesion was performed, confirming the initial histological diagnosis and identifying a BRAFV600E mutation that had not been detected in the previous biopsy. Because of recurrent/progressive disease after failure of standard therapy, vemurafenib was started at 5 years of age. At the start of targeted therapy, the measurable hypothalamic lesion measured 32.2 × 20.1 × 23.6 mm in the craniocaudal, anteroposterior, and laterolateral axes. Adherence was assessed by pharmacy refill records and pill counts. Reported toxicities included skin reactions, alopecia, hypercholesterolemia, neutropenia, and transient QT-interval prolongation, all grade 1 according to the Common Terminology Criteria for Adverse Events version 4.03 (CTCAE v4.03), system in current use at the time of the evaluation. A partial tumor response was achieved (14), with an approximately 55% reduction in tumor dimensions. Tumour response was assessed according to RANO criteria (14), which were used in clinical reporting at the time of evaluation. A partial response was reported in the available imaging records. After 10 years of vemurafenib treatment and radiological stability following the initial partial response, vemurafenib was discontinued.

Endocrine complications included central hypothyroidism and central adrenal insufficiency (CAI), both diagnosed at 6 months of age (few weeks after the tumor diagnosis) and requiring replacement therapy. Hydrocortisone was discontinued after 9 years of treatment, at the age of 9 years and 6 months of age, following a partial response to the low-dose ACTH stimulation test, defined by a cortisol peak between 16 µg/dl and 22 µg/dl. Central precocious puberty (CPP) was diagnosed at 7.5 years and treated with GnRH analogues for 4 years. After treatment withdrawal, pubertal progression remained slow; estradiol replacement was initiated at 12.8 years and Tanner stage III, and menarche occurred at 14 years (Tanner stage IV). **Figure 1** provides a schematic graphical representation of the timeline, illustrating the relevant data from the different episodes of care.

**Figure 1:**
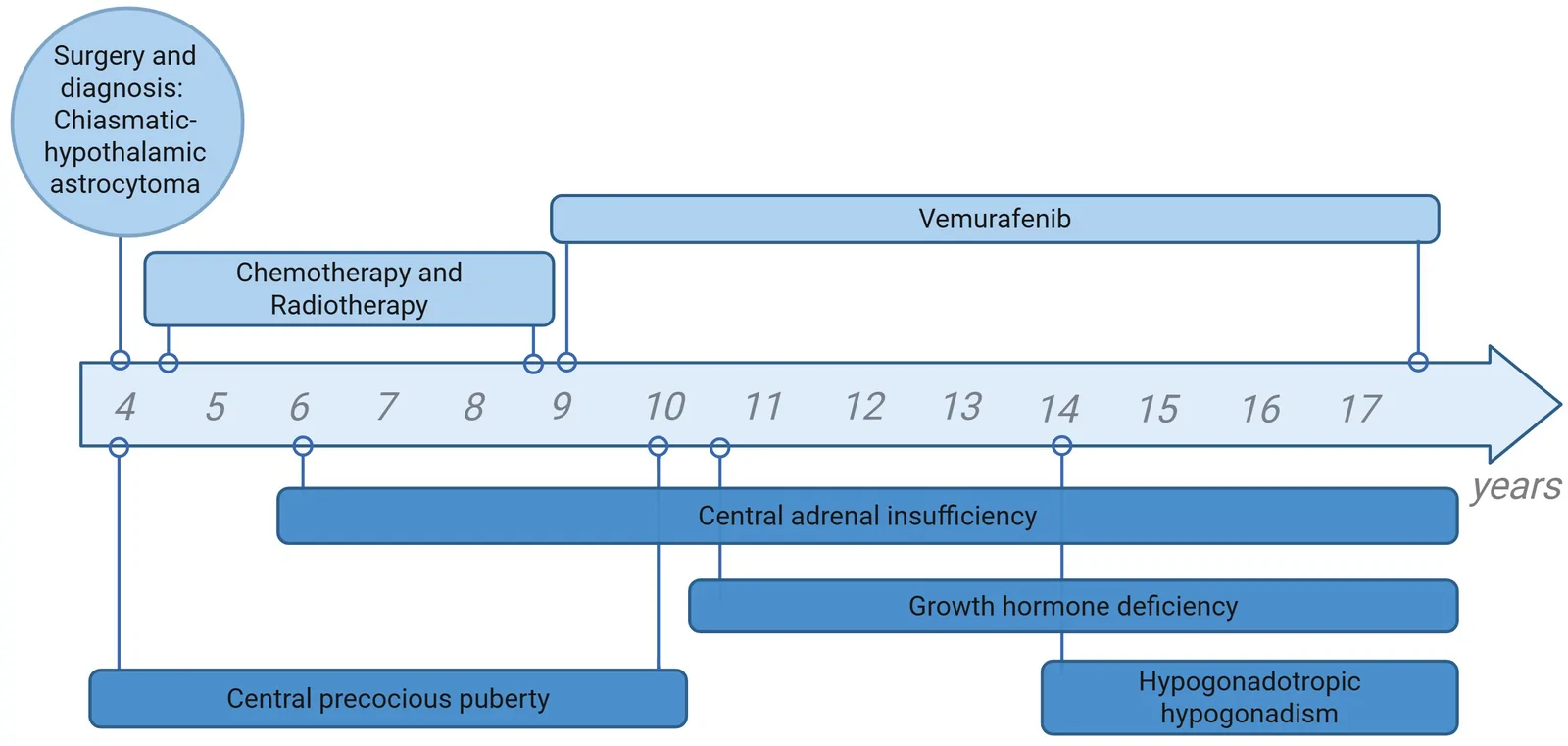
Schematic graphical representation of Case 1 timeline with relevant data from the episode of care.

Case 2: A male patient was diagnosed at 4 years of age with a chiasmatic-hypothalamic pilocytic astrocytoma. After partial surgical resection, initial chemotherapy was administered according to the SIOP LGG 2004 protocol. After 7 weeks, clinical and visual deterioration occurred, accompanied by an increase in lesion size on MRI. Because of the lack of response, a second subtotal resection was performed. Subsequent treatments included cisplatin and etoposide, of which 9 of 10 planned cycles were completed, vinblastine therapy, and locoregional radiotherapy to a total dose of 54 Gy (12). The number of fractions was not available in the external radiotherapy records. Histological review performed at our institute two years after disease onset confirmed the presence of a BRAFV600E mutation in previously analyzed biopsy specimens. Because of disease progression, vemurafenib was initiated at 9 years of age. A partial response was achieved according to RANO criteria (14), in used in clinical reporting at the time of evaluation. Adherence was assessed systematically by pharmacy refill records and pill counts. After 8 years of treatment, vemurafenib was discontinued at 17.5 years of age.

Endocrine complications included CPP as a presenting sign of tumor onset, treated with GnRH analogues until 10 years of age; CAI requiring hydrocortisone replacement; and hypogonadotropic hypogonadism (HH), treated with testosterone from 14 years of age, 11.5 years of bone age and Tanner stage of II. **Figure 2** provides a schematic graphical representation of the timeline, illustrating the relevant data from the different episodes of care.

**Figure 2:**
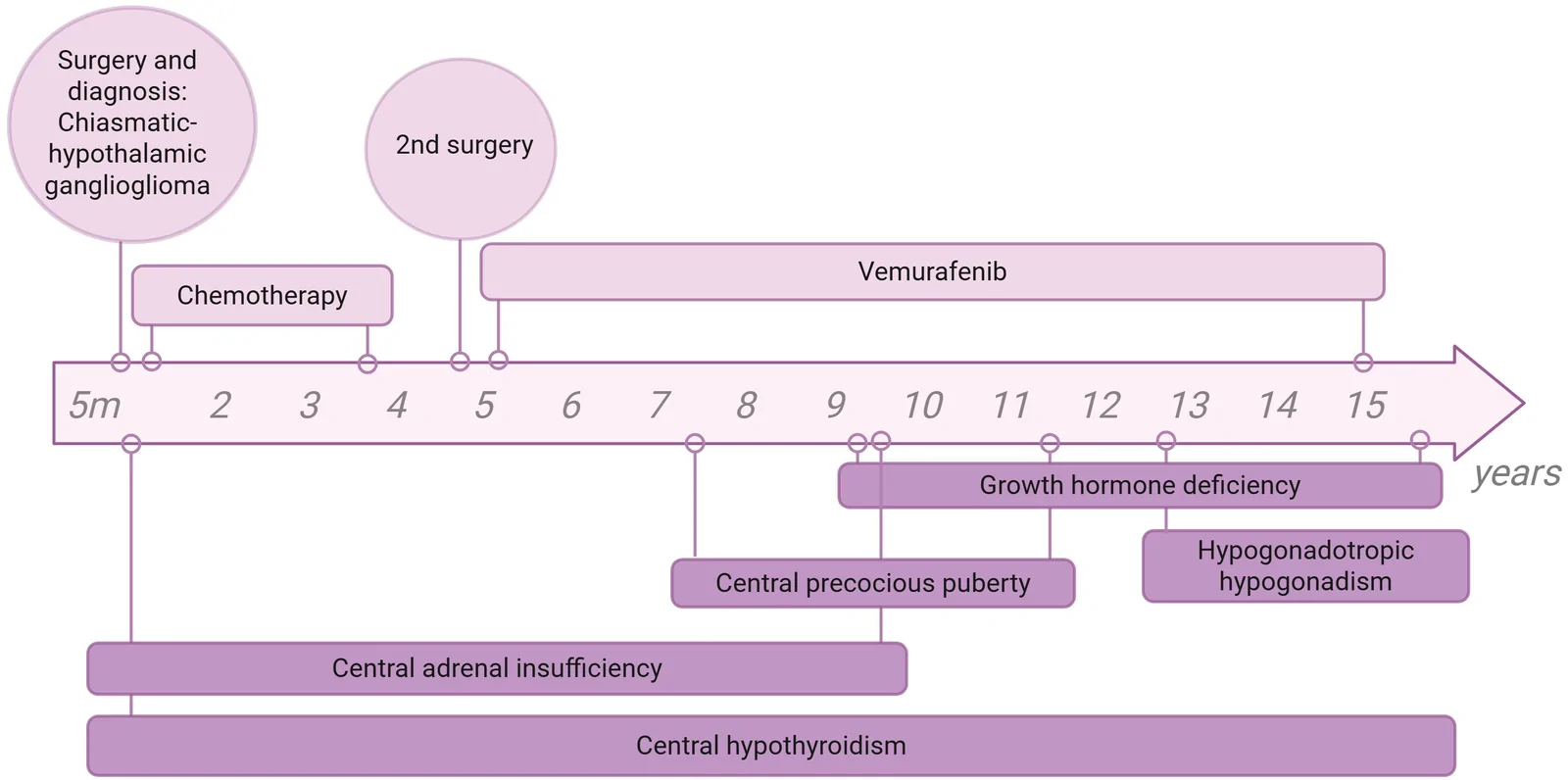
Schematic graphical representation of Case 2 timeline with relevant data from the episode of care.

### Diagnostic assessment, therapeutic intervention, follow-up, and outcomes according to the CARE guidelines

Case 1: GHD was clinically suspected already at 7 years of age because of progressive growth failure (**Table 1**), however, as she was on active oncological therapy, she was investigated biochemically later, at 9 years of age and Tanner stage of II. An arginine stimulation test showed a pathological GH peak of 2.43 ng/mL, confirmed by a glucagon stimulation test (GST; peak GH 1.65 ng/mL). Severe GHD was diagnosed in the setting of 4 years of radiologically stable disease on serial MRI, progressive height deviation, low bone mineral density (BMD), bone age aligned with chronological age, and coexisting metabolic impairment, including overweight, hyperinsulinemia, and hyperlipidemia. After multidisciplinary discussion and informed consent, GHRT was initiated during ongoing vemurafenib therapy. The decision was made after detailed counseling with the family regarding the absence of direct safety data for GHRT during active targeted therapy, the potential consequences of untreated severe GHD, and the planned close endocrine and neuroradiological surveillance.

**Table 1:** Clinical, auxological, metabolic, and DXA parameters approximately two years before GHRT initiation (T−2), at GHRT initiation (T0), and after approximately six years of GHRT (T6)*.

Parameter	Case 1			Case 2		
	T -2	T0	T6	T -2	T0	T6
Age (years)	7.0	9.2	15.4	8.7	11.0	17.4
Time on BRAFi (years)	2.0	4.2	10.4	0.0	2.0	8.4
Height SDS	-2.9	-3.6	-3.3	+1.7	+0.4	-0.3
Growth velocity (cm/y)	3.1	1.0	2.5	2.3	1.8	3.0
Growth velocity SDS	-3.2	-5.7	0.1	-4.1	-4.7	+3.1
BMI SDS (WHO)	+2.0	+1.6	+1.2	+4	+2.7	+3.0
Tanner stage	1	2	4	1-2	2	5
Hormone replacement therapy	levothyroxine hydrocortisone	levothyroxine hydrocortisone GnRH analogues	levothyroxine estradiol GHRT	hydrocortisone, GnRH analogues	hydrocortisone	hydrocortisone, testosterone, GHRT
TBLH BMD z-score	-2.9	-3.5	-3.2	0.5	-1.4	-1.2
L1-L4 BMD z-score	-1.7	-2.1	-3.2	-0.3	-0.9	-2.5
BMAD z-score	-1.4	-2.0	-2.7	-1.7	-1.5	-2.1
Fat mass (%)	54.2	54.6	51.0	53.6	48.0	47.2
Fat mass (kg)	9.1	11.3	22.8	29.6	23.9	43.8
Lean mass (kg)	7.7	9.4	21.6	35.7	25.9	48.9
Triglycerides (mg/dL; n.v. 30-160)	163	175	134	110	127	132
Total cholesterol (mg/dL; n.v. 80-180)	303	267	241	178	230	196
LDL cholesterol (mg/dL; n.v. 0-130)	225	195	186	124	158	151
HDL cholesterol (mg/dL; n.v. 55-100)	43	39	48	42	45	36
IGF-1 SDS	-0.7	-3.0	+1.5	2.3	-0.4	+0.95

*T6 refers to the last available follow-up approximately six years after GHRT initiation: 6.2 years in Case 1 and 6.4 years in Case 2. Interpretation of growth and DXA outcomes should consider pubertal timing and sex-steroid replacement: Case 1 received GnRH analogue therapy followed by estradiol replacement from 12.8 years of age; Case 2 received GnRH analogue therapy followed by testosterone from 14 years of age. At T−2, Case 2 had not yet started BRAFi therapy.

SDS, standard deviation score; BMI, body mass index; WHO, World Health Organization; TBLH, total body less head; BMD, bone mineral density; L1-L4, lumbar vertebrae 1-4; BMAD, bone mineral apparent density; n.v., normal values; LDL, low-density lipoprotein; HDL, high-density lipoprotein; IGF-1, insulin-like growth factor 1. Height, BMI, growth velocity, BMD, BMAD, and IGF-1 values are reported as SDS/z-scores according to the reference standards cited in the text. Lipid reference ranges are shown in parentheses where available. Lipid reference ranges are laboratory-specific; LDL cholesterol interpretation followed local pediatric laboratory standards.

no additional correction are applied to TBLH BMD z-score and lumbar spine BMD z-score, apart from adjustment for age and ethnicity. BMAD z-score provides an adjustment for vertebral size in the interpretation of lumbar spine BMD.

Treatment was started at two-thirds of the standard dose, 0.021 mg/kg/day, and titrated over two months to 0.024 mg/kg/day corresponding at 4.9 mg/m²/week, with subsequent dose adjustment according to growth response and IGF-1 levels. IGF-1 increased from -3.0 SDS (15) before GHRT initiation to +1.5 SDS (15) at the last follow-up. This value remained within the recommended age-, sex-, and pubertal stage-adjusted range for GH replacement therapy, generally ≤+2 SDS (11), and was interpreted in the context of Tanner stage IV and ongoing estradiol replacement. During GHRT and vemurafenib co-treatment, the patient underwent quarterly clinical and biochemical assessments and semiannual MRI surveillance. Serial imaging showed sustained radiological stability throughout the reported period. At 14 years of age and Tanner stage of III the patient developed insulin resistance. Given attainment of near-adult height and recent discontinuation of vemurafenib, GHRT was stopped at 15 years and 5 months, bone age was 14.5 years and Tanner stage IV.

Height remained severely reduced but relatively stable, from -3.6 SDS at baseline to -3.3 SDS at T6, corresponding to near final height, with a final deviation of -1.3 SDS from target height, which was -2 SDS. Growth velocity gradually improved from 1.0 cm/year (corresponding to -5.7 SD) at baseline to 6.5 cm/year (1.2 SD) after one year of treatment and 6.1 cm/year after two years. At T6, growth velocity was 2.5 cm/years (corresponding to +0.1 SD) at T6. Bone age was delayed by 3 years and 7 months at baseline and progressively caught up with chronological age, with a residual delay of approximately 1 year at T6. BMI SDS (16) and DXA-derived fat mass percentage decreased slightly, from 54.6% to 51.0%. Bone mineral content (BMC) increased; however, lumbar spine BMD z-score declined from -2.1 to -3.2, and bone mineral apparent density (BMAD) z-score declined from -2.0 to -2.7. By contrast, total body less head (TBLH) BMD z-score improved slightly, from -3.5 to -3.2. Metabolic parameters improved, with reductions in triglycerides, total cholesterol, and LDL cholesterol, and an increase in HDL cholesterol. No tumor progression or secondary malignancies were detected during 6.2 years after GHRT initiation, corresponding to the period of concomitant GHRT and vemurafenib exposure described in this report.

Case 2: GHD was diagnosed at 10.5 years of age, because of progressive growth failure (**Table 1**), with a bone age delay of 6 months and Tanner stage I. An arginine stimulation test showed a pathological GH peak of 2.25 ng/mL, confirmed by GST, with a peak GH of 1.43 ng/mL. Given severe GHD with progressive growth impairment and metabolic alterations, a multidisciplinary decision was made to initiate GHRT despite ongoing antitumor therapy, in the context of 2 years of radiologically stable disease. The decision was made after detailed counseling with the family regarding the absence of direct safety data for GHRT during active targeted therapy, the potential consequences of untreated severe GHD, and the planned close endocrine and neuroradiological surveillance.

GHRT was initiated at 0.01 mg/kg/day, corresponding to 2.5 mg/m²/week and titrated over two months to 0.021 mg/kg/day corresponding to 5.4 mg/m²/week. The dose was subsequently reduced to 2.63 mg/m²/week according to IGF-1 levels and weight. At the last follow-up, IGF-1 was +0.95 SDS (15).

During GHRT and vemurafenib co-treatment, the patient underwent regular clinical, biochemical, and neuroradiological follow-up. Although GHRT was initiated before advanced pubertal development, the long-standing hypothalamic-pituitary damage and complex treatment history may have limited the potential for catch-up growth. Growth velocity improved, from 1.7 cm/year (corresponding to −4.7 SD) at baseline to 4.2 cm/year after one year of treatment and 3.8 cm/year (-1.9 SD) after two years, reaching 3.0 cm/year (corresponding to 3.1 SD) at T6. Bone age was delayed by 6 months at baseline and progressively caught up with chronological age, with a residual delay of approximately two years at T6. Bone age was delayed by 6 months at baseline and progressively diverged from chronological age, reaching a delay of approximately 2 years at T6. Final height remained aligned with target height, with a final deviation of -0.2 SDS at T6. At T6 he was 17.4 years old with bone age delay of two years and adult pubertal status. However, weight and BMI increased markedly, reaching 100 kg and +3 SDS according to WHO criteria, respectively (16). Severe obesity, likely related to hypothalamic damage from the tumor itself and surgical treatment, was managed with nutritional counseling and dietary intervention, with only partial benefit. No pharmacological treatment for hypothalamic obesity was initiated during the reported period. Further hypothalamic assessment was subsequently scheduled.

DXA confirmed persistently elevated fat mass percentage. Although BMC increased, lumbar spine BMD z-score declined from -0.9 to -2.5, whereas TBLH BMD z-score remained within the low-normal range for age and sex. Lipid profile improved, while triglycerides remained stable. No tumor progression or secondary malignancies were detected during 6.4 years after GHRT initiation, corresponding to the period of concomitant GHRT and vemurafenib exposure described in this report.

Overall, the female patient showed greater improvement in metabolic parameters, whereas linear growth remained limited. The male patient developed severe obesity, likely reflecting the dominant contribution of hypothalamic involvement (17). In both patients, BMC increased, while lumbar BMD z-scores declined, suggesting that skeletal growth and changes in vertebral size may have outpaced mineral accrual. These bone findings should be interpreted in the context of pubertal timing, hypogonadism, sex-steroid replacement, hypothalamic disease, prior oncologic treatments, and body-composition changes. **Table 1** summarizes key clinical, metabolic, and DXA-derived parameters two years before GHRT initiation, at GHRT initiation, and after approximately six years of GHRT.

## Discussion

Current guidelines recommend considering GHRT in childhood cancer survivors (CCS) with confirmed GHD only after an adequate period off oncologic therapy and in the presence of stable disease, following multidisciplinary evaluation (11). Accordingly, GHRT use during active anticancer treatment remains controversial, mainly because of concerns regarding tumor progression, recurrence, and secondary malignancies. Large cohort studies in CCS, including a French series of 2,852 survivors, have not shown an increased overall risk of secondary neoplasms associated with GHRT (18). An increased incidence of meningiomas has been reported in some survivor cohorts, although this appears to be primarily related to prior cranial radiotherapy rather than to GHRT itself (19). However, these data mostly derive from patients in remission or after completion of oncologic therapy and therefore cannot be directly extrapolated to children receiving GHRT during ongoing targeted antitumor treatment.

The increasing use of molecularly targeted therapies, particularly BRAF and MEK inhibitors, has changed the management of recurrent or progressive pLGG but has also created new long-term endocrine and metabolic challenges. In selected patients, prolonged vemurafenib treatment may be required to maintain tumor control, exposing children to chronic treatment-related toxicities that require systematic monitoring. In adult melanoma studies, including the BRIM-3 trial, vemurafenib was associated with adverse events such as rash, arthralgia, alopecia, photosensitivity, nausea, and cutaneous malignancies (20). Dyslipidemia, including increased triglycerides, total cholesterol, and LDL cholesterol, has also been reported during both short- and long-term exposure to BRAF inhibitors (20–22).

In this context, the decision to initiate GHRT during prolonged vemurafenib therapy requires individualized assessment. Potential benefits on growth, body composition, lipid metabolism, and bone health must be weighed against the uncertainty surrounding GH replacement during active oncologic treatment. Evidence from patients with RASopathies, such as Noonan syndrome, may provide only indirect and hypothesis-generating reassurance. Although these patients have underlying MAPK pathway dysregulation and increased baseline cancer susceptibility, available reports have not identified a clear oncogenic safety signal with GHRT (23). Nevertheless, this setting is biologically and clinically distinct from children with active BRAFV600E-mutant pLGG receiving BRAF inhibitor therapy, and direct extrapolation is not appropriate.

In the present cases, GHRT was initiated only after a long-standing clinical suspicion of GHD had been confirmed by biochemical evidence of severe GHD, sustained radiological stability had been documented, and a multidisciplinary consensus had been reached. Treatment was introduced cautiously, using conservative dosing and regular IGF-1 monitoring as part of quarterly endocrine follow-up, with dose adjustment intended to avoid sustained supraphysiological IGF-1 exposure. Importantly, the indication for GHRT was not limited to impaired linear growth, but also included the broader clinical consequences of untreated severe GHD, including adverse body composition, metabolic impairment, and reduced bone mineral status.

The auxological response was clinically modest in both patients. Height SDS remained severely reduced in the female patient, although growth velocity improved. In both patients, growth velocity SDS increased substantially after GHRT initiation; however, this translated mainly into growth stabilization rather than marked catch-up growth. These findings suggest that, in children with long-standing hypothalamic-pituitary damage and complex oncologic histories, GHRT may contribute more to growth stabilization than to marked catch-up growth, particularly when treatment is started late or when pubertal progression and sex-steroid replacement influence residual growth potential.

The metabolic outcomes were also heterogeneous. The female patient showed improvement in BMI SDS, fat mass percentage, and lipid profile, suggesting a potential favorable effect of GHRT on body composition and cardiometabolic risk. In contrast, the male patient developed severe obesity despite treatment, although lipid parameters partially improved. This divergent response likely reflects the dominant contribution of hypothalamic involvement, prior treatments, pubertal status, and individual differences in energy balance. Therefore, while GHRT may have contributed to metabolic improvement, particularly in lipid profile, these observations cannot be separated from the effects of hypothalamic injury, vemurafenib exposure, lifestyle factors, pubertal development, and concomitant endocrine replacement.

Bone outcomes require particularly cautious interpretation. In both patients, bone mineral content increased during follow-up, consistent with skeletal growth and increasing body size. However, lumbar spine BMD and BMAD z-scores declined, indicating that mineral accrual may not have kept pace with changes in bone size, pubertal development, or body composition. This pattern may reflect the combined effects of severe GHD, hypogonadism or delayed/altered pubertal progression, obesity, reduced mobility, prior oncologic treatments, and, in the male patient, previous radiotherapy. These findings underline that GHRT alone may be insufficient to normalize bone health in children with complex hypothalamic-pituitary morbidity and that bone outcomes should be interpreted alongside pubertal status, sex-steroid replacement, vitamin D status, calcium intake, physical activity, and DXA-derived measures adjusted for body size. Overall, the substantial complexity of both cases, including multiple comorbidities and potential confounding from induced pubertal development, sex-steroid replacement, and hypothalamic obesity, must be taken into account when interpreting the effects of GHRT on bone and body composition, as these factors cannot be fully disentangled from the effects of GHRT. To provide a better overview of the clinical trajectory, auxological, DXA and metabolic data from approximately two years before GHRT starting are provided in **Table 1**.

From an oncologic perspective, no tumor progression or secondary malignancies were detected during 6.2 and 6.4 years of concomitant GHRT and vemurafenib exposure. This prolonged observation is reassuring at an individual-patient level, particularly because both children had previously progressive BRAFV600E-mutant pLGG requiring multiple lines of treatment. However, these findings cannot establish oncologic safety. The absence of progression may reflect pre-existing tumor stability under vemurafenib, the biology of the individual tumors, or other unmeasured factors. Therefore, these cases support the possibility that GHRT can be administered under strict surveillance in selected patients, but they do not remove the need for caution.

Overall, these two cases suggest that GHRT during BRAFi treatment may be considered in highly selected patients with severe confirmed GHD, sustained radiological stability, and close multidisciplinary surveillance. The observed benefits appeared more evident for metabolic profile and growth stabilization than for catch-up growth or bone mineral recovery. Larger prospective studies are required to evaluate safety, define optimal timing and dosing, identify predictors of response, and clarify which outcomes should guide treatment decisions in this specific population.

This report has limitations, but it also provides clinically relevant information in an area where direct evidence is scarce. Its strengths include the description of two highly selected pediatric patients with severe confirmed GHD treated with GHRT during ongoing BRAF-inhibitor therapy, the prolonged concomitant follow-up of more than six years, and the detailed assessment of endocrine, auxological, metabolic, DXA-derived, and oncologic outcomes. The multidisciplinary decision-making process, conservative GHRT dosing, IGF-1 monitoring, and close surveillance may be useful for clinicians facing similar scenarios.

Nevertheless, the findings must be interpreted with caution. The sample size is limited to two patients, and no causal inference regarding efficacy or oncologic safety can be made. Both patients had complex multimodal treatment histories and multiple endocrine comorbidities that may independently affect growth, metabolism, and bone health. Detailed oncologic data were not reported in full because of the inherent limitations of the retrospective study design and the incomplete availability of radiotherapy and old imaging data, particularly for one patient. Interpretation of metabolic and DXA-derived outcomes is also limited by the absence of a comparator group and by incomplete control for lifestyle, pubertal, nutritional, and body-composition variables. These observations should therefore be regarded as hypothesis-generating and require confirmation in prospective multicenter cohorts.

## Patient perspective

A formal patient or family perspective was not collected for this case series. However, in both cases, the decision to initiate GHRT during ongoing vemurafenib therapy was discussed in detail with the families, including the absence of direct safety data for GHRT during active targeted therapy, the potential consequences of untreated severe GHD, and the planned close endocrine and neuroradiological surveillance.

In these two pediatric patients with BRAFV600E-mutant chiasmatic-hypothalamic pLGG, GHRT was initiated during ongoing vemurafenib therapy after biochemical confirmation of severe GHD, documented radiological stability before GHRT initiation, and multidisciplinary agreement. A conservative dosing strategy was used, with regular IGF-1 monitoring and dose adjustment to avoid sustained supraphysiological exposure.

During 6.2 and 6.4 years of concomitant GHRT and vemurafenib exposure, no tumor progression or secondary malignancies were detected. Although reassuring at the individual-patient level, this observation cannot establish oncologic safety. Clinical outcomes were heterogeneous: benefits appeared more evident for lipid profile and growth stabilization than for marked catch-up growth, while bone outcomes remained complex, with persistent or worsening lumbar DXA-derived impairment despite increased bone mineral content.

These cases suggest that GHRT during BRAFi therapy may be considered only in highly selected patients with severe confirmed GHD and close endocrine-oncologic surveillance. Prospective multicenter studies are needed to evaluate safety, define optimal timing and dosing, and clarify which auxological, metabolic, skeletal, and oncologic outcomes should guide treatment decisions in this specific population.

## Data Availability

The original contributions presented in the study are included in the article/supplementary material. Further inquiries can be directed to the corresponding author.
